# Music in an Emergent History of Psychology

**DOI:** 10.1007/s12124-023-09778-9

**Published:** 2023-06-03

**Authors:** Sven Hroar Klempe

**Affiliations:** Dept. of Psychology, Norwegian University of Science and Technology (NTNU), Trondheim, Norway

**Keywords:** History of psychology, Music, Experimental psychology, The origin of psychology, Psychology in the sixteenth century, Castellani, Freigius

## Abstract

From a historical perspective, ‘psychology’ can be studied from an abundance of angels. Thus, a selected perspective requires some historiographical reflections, but also a conscious awareness of the actual chosen terms that are at stake. In this study, the historiographical perspective follows an emergent understanding of the history, which implies that the actual chosen terms are dynamically contributing to a web of terms, in which all of them may change in more or less unpredictable directions. In line with this, the aspect of music is consciously chosen, as it probably is one of the most ignored aspects of psychology in historical research. Thus, the findings in this study reveal that music as the ’direct factor’ played an overarching role in the nineteenth centuries experimental psychology, but also that the changes in the understanding of music in the early sixteenth century is comparable with the changes the understanding of the soul underwent along with the introduction of the neologism ‘psychology’. In the understanding of both music and the soul the sensational aspects replaced the mathematical.

Since the historian Roger Smith (1988) problematized the aim of presenting a history of psychology, historiographical issues have been at stake in psychology. By doubting psychology having a subject, Smith demonstrated that it is hard to find unmistakable definitions of the most important key terms in such a history, but also that psychological terms seldom have specified objects that are naturally given. The term ‘psychology’ itself is the best example, as this is hard to give an overall valid definition. Scholars, therefore, try to avoid talking about ‘the history of psychology’, and prefer this to be replaced with ‘historical psychology’ (Danziger, 2010). Here, therefore, I will bring in an aspect, which may reveal some new perspectives on the development of psychology and its origin, namely the aspect of music.

## Some Historiographical Premisses

In a paper from 2003 Danziger (2010) elaborate on how an historical psychology is to be envisaged. As a point of departure, it is negatively understood, as it should not investigate “its subject matter as though it belonged to an ahistorical human nature” (Danziger, 2010, p. 129). This implies that matters in psychology are dominated by entities that may change along the history. In contrast, historical psychology tries “to question contemporary psychological concepts in the light of historical evidence” (p. 129). This is a kernel sentence, as it highlights two coordinated factors that characterize historical psychology: historical evidences and concepts. In line with this, Danziger refers to Michel Foucault, who belonged to the French school of annals, which first of all focused on documents. A document exemplifies nicely the two different directions a historical evidence may point, namely to a physically existing object and a textual content. Whereas the document itself can be regarded as an unequivocal entity, the textual content is equivocal and may generate many different sorts of narratives.

In line with this, one may easily well get lost in the abundance of narratives just a simple document may generate. Thus there is a need for evaluations, arguments and corrections. Hence Danziger refers to another important term, namely ‘genealogy’ borrowed from Nietzsche (2012), who argues: “We don’t know ourselves, we knowledgeable people – we are personally ignorant about ourselves” (p. 1). By focusing on the origin (Herkunft) and our prejudices (moralisches Vorurteile), a *diachronic* perspective forms a *tool* by which we may reveal the hidden aspects of human discourses.

A third aspect is *change*. An origin produces immediately a difference between past and present. This is especially true when it comes to psychological terms, as both Danziger (2010), and Graham Richards (2002) have stated. Terms like memory, emotions, learning, personality etc. are all terms that have changed along the history. However, this perspective forms also a core aspect of the thesis on the arbitrary sign (Saussure 2011), which says that all languages change in line with the use of them. The meaning of a term is not given by its reference, but a result of the fact that language is a self-constituting system, in which the opposition between terms produces the meaning of them. This is the radical turn in linguistics. Saussure faced the challenge of explaining why language changes continuously in the long run, but at the same time appears as fixed and stable. Blumenthal (1973) documented a connection between Wundt and Saussure, and Wundt (1902) also mentions the role of oppositions in affective qualities (p. 37). The thesis of the arbitrary sign is not limited to language, but represents an overall mode of thinking that permeated French structuralism (Piaget, 1973), but also some aspects of post-structuralism (Brochier, 1978).

Thus, to have a historical perspective is to focus on changes. However, the diachronic perspective is never complete when a systematic synchronic perspective is left out. This has not attracted too much attention, but historical psychology opens up for this, as it focuses on the contemporaneous uses of terms to avoid ‘Whigishing’ the history, which may end up with ‘presentism’.

Some of the systematic aspects in historiography are given by hermeneutics, which became crucial when theologians like Martin Luther for example began to problematize the Church’s traditional understanding of the Christian tenets and dogmas in the fifteenth and sixteenth centuries. Before this time texts were understood as if the content of a text is unambiguous. This changed when Luther saw the Bible telling just one continuous story with a certain perspective, into which every detail of the text should be interpreted. This emphasis on interpretation was enforced 200 years later when Giambattista Vico launched the *verum factum* principle, which opened up for more variety of interpretations of a text (Tateo, 2017). Wilhelm Dilthey expanded the content of hermeneutics by working out a systematic understanding of history by means of this principle and hermeneutics (Wind, 1976).

When date and place is decided, the hermeneutic process starts with placing the object within a geographical and historical context. This implies a production of alternative interpretations, which should end up with a likely understanding. Likeliness is a *sine qua non* in historical research. The arguments may have different forms, but the result is a construction of narratives that can be associated with the actual object.

There will always be produced new narratives seeing the same event from different angels. This makes the term ‘genealogy’ crucial, which Nietzsche’s analysis of morals demonstrates (Nietzsche 2012). There are many types of moralities, and in this perspective, a term’s equivocality and ambiguity have to be taken into account. The challenges we face in historical research are not restricted to the author, but even as much includes the reader. The reader has to go through the same critical process, which implies that the reader has to take into account the criteria on which the historical presentation is based. Nietzsche’s authorship can for example be read as if he was a precursor for Nazism, but it can also be read from an opposite perspective, namely that he uncovered and indirectly warned against tendencies that actually led to Nazism.

In line with this, changes are present on many different levels at the same time. The fact that a term’s meaning change is based on several factors, which all point in the direction of the thesis of the arbitrary sign. This thesis states that the sign system is a self-constitutive system, in which each element in the system has impact on the other elements in terms of standing in opposition to each other. This is a transitive system in the sense that if one element stands in opposition to another element, the other element stands in opposition to a third element. However, if the latter opposition changes, the first opposition will also go through a sort of change as well.

## Different Perspectives on Thinking

One example that may illustrate this can be the term ‘thinking’. We may trace the discussion about the meaning of this term back to early modernity when the validity of thinking in Western philosophy was evaluated on its clearness and obvious truth. Descartes (Descartes, 1641/1960, part III), for example, used this criterion of clearness to proof God’s existence, as he stated that all ideas have a cause. Our ideas are normally a result of our sense impressions or our subjective imaginations. However, any person’s ideas about the perfect and infinity cannot have its origin in external or internal impressions, as there are nothing that is perfect or infinite in a human or the world. Thus, these ideas must be inherited ideas that have their origin in something that in fact is perfect and infinite, which is God. Hence, the clearness of these ideas, therefore, proofs that God must exist. This argument and the critics of it demonstrated the turmoil in philosophy in early modernity, which was about throwing theology out from philosophy and replace theology with psychology (Klempe, 2020). After psychology had invaded metaphysics in German rationalism when Christian Wolff explicitly included it and consequently redefined metaphysics in the 1730ies, Immanuel Kant saw it as his task to throw psychology out of philosophy again. In this achievement, he not only developed a critical philosophy, but also defined thinking as being nothing else than a rational use of terms. The consequence of this was that thinking and the basis of it was given by language itself.

## Music as the Direct Factor

In the wake of Kant’s philosophy, there were no clear distinction between philosophy and psychology. This lack of distinction counts for both the empiricists and the idealists, as the empiricists focused on sensation and the idealists focused on subjectivity. Yet when experimental psychology began to take form, the Kantian tendency to entrench thinking processes in language was challenged. It is hard to name the founder of experimental psychology, as psychological experiments can be traced back to the middle of the eighteenth century (Klempe, 2020). However, psychological experiments achieved a very peculiar profile hundred years later when Fechner launched the concept of ‘experimental aesthetics’ (Fechner, 1871/1978). The aim of these experiments was to focus on sensational impressions and examine their impact on the mind before they had been transformed into clear ideas that could be expressed through words. With this aim, music became of a certain interest, as it is “von Vorstellungsassociationen unabhängig” (Fechner, 1871/1978, p. 150) – independent of associations that are related to specified ideas. Thus, to focus on the direct factor in experimental psychology was highly related to an interest in exploring the aspects of the mind that lies beyond language.

This idea of music as the direct factor is an underestimated and overlooked factor in experimental psychology, yet spread among some few scientists in the North-Eastern Preussen. The physicist, physiologist and polymath Herman Helmholtz adopted this idea and formulated it as a premise for his investigation of sensation (Helmholtz, 1874/1954, see p. 2–3). Wilhelm Wundt brought this a step further when he established and developed his laboratory, in Leipzig. He furnished it with almost the same equipment as Helmholtz’ acoustic laboratory. Thus the difference between the two laboratories was not related to the equipment, “but *different points of view*” (Wundt, 1902, p. 2, original italics). Whereas physics and natural sciences focus on the “*objects of experience*” (p. 3, original italics), psychology focuses on the “*experiencing subject*” (p. 3, original italics). Wundt was also guided by the idea of music as the direct factor, as his acoustic laboratory was equipped with about 350 tune forks, among other things, and most of the research he referred to took place in the acoustic laboratory, and not so much in the visual laboratory (Klempe, 2011).

This perspective on experimental psychology was followed up by many German psychologists in the latter part of the 19th century, and not least by the Berlin based experimentalist Carl Stumpf. By focusing on the relationship between consonances and dissonances he demonstrated experimentally that the perceived differences between them were completely relative. Relativity represents a type of non-verbal cognition that belongs to the higher cognitive functions (Stumpf, 1883, 1890).

The importance of the relative and the focus on music were both adopted and followed up by the Gestalt psychologists. Christian von Ehrenfels’ article on ‘Gestalt qualities’ (Ehrenfels, 1890/1988) referred to a broad discussion among scholars who pursued the question “What is a melody?” (Ehrenfels, 1932/1988). The answer Ehrenfels gave had wide consequences, as transposition demonstrates the fact that an isolated pitch does not count, but instead the relations – the intervals – *between* the pitches.

## Thinking and Language

Although Gestalt psychology is not directly associated with Carl Stumpf, the three pioneers Wolfgang Köhler, Kurt Koffka and Max Wertheimer were all more or less students of him (Ash, 1998). In Wertheimer’s presentation (Wertheimer, 1925/1967) Gestalt psychology was summarized in this way:

There are wholes, the behaviour of which is not determined by that of their individual elements, but where the part-processes are themselves determined by the intrinsic nature of the whole. It is the hope of Gestalt theory to determine the nature of such wholes. (Wertheimer 1925/1967, p. 2.)

The Gestalt was not only regarded as a psychological factor, but formed also an epistemological premise. Thus thinking processes was regarded as going far beyond language because the perceived relations between composite elements are normally impossible to put into words.

Vygotsky’s thesis on *Thinking and Speech* (Vygotsky, 1987) can be understood in line with these observations. The key to understand this is the nature of what he calls ‘the inner speech’. The latter is not a well-formed use of language but rather the opposite. It is “a process that involves the evaporation of speech in thought” (p. 257). Moreover, the inner speech has its origin in the egocentric speech, which is ungrammatical and almost not understandable. The inner speech subverts well-formed language, and this process opens up for pure thinking. The reversal “is a process of transforming thought into word; it is the materialization and objectivization of thought” (p. 257). Consequently, “[t]hought is always something whole, something with significantly greater extent and volume than the individual word” (p. 281). Thus thinking may contain words, but the most essential aspect of thinking is this wholeness of the elements, which is provided by the relations between them.

This is comparable with Saussure’s thesis of the arbitrary sign (2011). As mentioned, a term does not acquire its meaning with its reference, but by standing in opposition to other signs. Thus, when thinking in terms, not the words themselves provide meaning, but rather the relations between them. Hence, by means of the thesis of the arbitrary sign, it is possible to demonstrate that even thinking in concepts is based on processes that go far beyond language.

This turned into a complete opposite understanding when Noam Chomsky (Chomsky, 1957/1975) presented his ideas about how to understand syntactic structures in language with a precision comparable with mathematics on a seminar at MIT the 11th Sept. 1956 (Miller, 2003). According to Miller, the cognitive science as a research field was more or less born that day. There is no doubt that cognitive psychology did get a boost through and after the 1950ies (Neisser, 1967), and it has dominated all Western psychological research for more than half a century. Chomsky presented crucial principles for this research, which included seeing the linguistic capacity as a basis for human cognition in general. In this perspective, the cognitive revolution in psychology more or less reintroduced a Kantian perspective on thinking, as it regarded a logical and rational use of language as forming the basis for all human thinking. Because this cognitive perspective has dominated almost all Western psychology since the 1950ies, it has been very different from how the pioneers in experimental psychology, the pioneers in Gestalt psychology, and the pioneers in Russian psychology before and after the turn of the previous century wanted to conceptualize thinking.

## Some Historiographical Consequences

The most important historiographical aspects of this example are at least two: (1) the content of a concept used in psychology is changing, and (2) it is hard to tell if the changes represent a progress. Yet a third aspect should not be underestimated, namely that (3) within a certain position, there are different and sometimes contradictory perspectives involved. This was the case when for example Wundt and Stumpf clashed in a discussion about the role of introspection, when Köhler, Koffka and Wertheimer rejected Ehrenfels’ concept of ‘qualities’, or when Piaget (1973) accommodated some of Vygotsky’s criticism of him and adopted a more Saussurian stand by defining the mind as a system that constitutes itself. Thus there is an abundance of factors, which blur the different tendencies and schools and make it difficult to talk about them as clear univocal directions in the history of psychology. Theoretical positions exist and pull in different directions at the same time. This kaleidoscopic emergence of more or less viable ideas in psychology are in line with the subject’s nature. The mind and the human nature may not yet be fully understood. They must be seen in the perspective of this insurmountable complexity. Thus, the aspect of an undefeatable complexity must form a kind of underlying premise for all approaches in psychology. However, this does not contradict the fact that we can trace the emergence of concepts that have been associated with the field of psychology.

## Music and the Emergence of ‘psychology’

It is in this perspective, tracing the emergence of ‘psychology’ in the sixteenth century is of great interest. The term is full of contradictory tendencies, which reflects the turmoil in Europe at the same time. The important historian Paul Mengal (2005) focuses on Rudolf Goclenius the elder (1547–1628), who published an anthology in 1590 with the term ‘psychology’ mentioned in the title. Several important traces were uncovered in Mengal’s study. A couple of he most important of them should be (1) the distinction between mental faculties of the soul (animus) and the free, eternal and spiritual soul (anima). In the ancient Greek this distinction had been obvious, but after the church father Isidor of Seville merged the two parts, it had been absent for almost thousand years (Vidal 2011). The other was (2) a distinction between those who thought the whole mankind has inherited the soul directly from Adam and his fall (traducianism), and those who thought that each soul is created separately for each individual (creationism) (Mengal, 2005). The latter is an important discussion as creationism underlines the tendency to focus on the individual. However, traducianism is slightly comparable with the role genes play in current medicine and psychology.

**Fig. 1:**
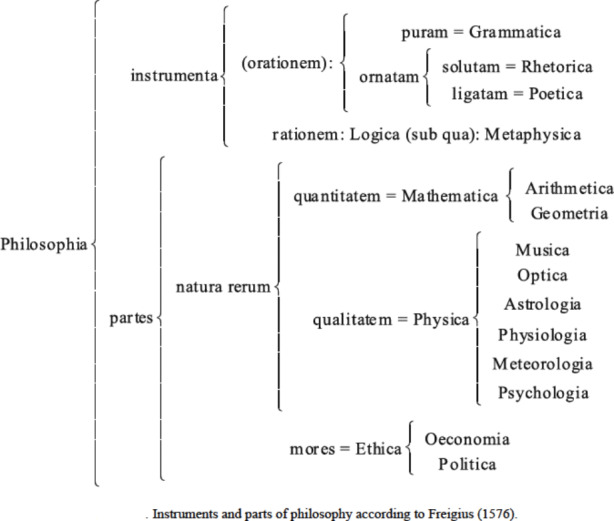
in: Sven Hroar Klempe: Music in an emergent history of psychology. (Reproduced from Luccio 2013, p. 7)

For a long time, it was unclear when the term ‘psychology’ exactly appeared in Western psychology, although all agreed upon its appearance in the sixteenth century. In 1964, a Jugoslavian librarian (Krstic, 1964) published an article, which documented that the Croatian Humanist Marko Marulić had published something entitled “*Psichiologia, de ratione animae humanae*”. Since Marulić died in 1520, it must have been written before that year. However, we have not the publication itself, just this title, which underlines the fact that the term refers to the human soul. Thus, it is of great interest to see how the soul and the term ‘psychology’ were conceptualized back then. There are several sources that can be focused on from this perspective. One is the writing of Philipp Melanchthon, who for a long time was regarded as the one that first used the term ‘psychology’. However, he never used the term, but he published some lectures on Aristotle’s “On the Soul”. Another scholar that is of crucial interest in this perspective is the German philosopher Johann Thomas Freigius (1543–1583). He is following up the more than thousand years old tradition of categorizing the different sciences or ‘arts’, into seven – the so-called Seven Liberal Arts. These seven sciences are again divided into two: *trivium*, consisting of Grammar, Rhetoric, Dialectics; and *quadrivium*, consisting of Arithmetic, Music, Geometry, and Astronomy. Freigius, however, constructed a scheme that was much more subtle and nuanced than the old categorization, although the ground structure of the seven liberal arts are traceable still (Fig. 1).

The big change that took place along with the sixteenth century is that ‘*physica’* is separated from ‘*mathematica’*. In line with this, quality became separated from quantity. The latter represented a complete turmoil, as the seven liberal arts did not have this distinction. *Quadrivium* included originally all the arithmetic sciences. This was specified in different ways during the history, but at around year 1000, for example in a textbook (*Didascalion*) written by the Augustinian influenced medieval scholar Hugo or Hugh of St. Victor (Sundberg 2002), *Quadrivium* was divided into ‘multitudes’ (*multitudo*) and ‘magnitudes’ (*magnitudo*). The former consisted of mathematics as such (*mathematica per se*), which is arithmetic, but also mathematics in relation (*mathematica ad aliquid*), which is music. In the group of magnitudes, we find the immobile mathematics (*mathematica immobilis*), which is geometry, but also moving mathematics (*mathematica mobilis*), which is astronomy. This organisation of the arts or sciences indicates that *Quadrivium* was regarded as containing different systems of quantities until the sixteenth century. This changes fundamentally when Freigius defined both music and astrology as qualities instead of quantities. The remarkable thing is that psychology is also included in this category, yet appears in this scheme as if it came from nowhere.

## The Castellani Manuscript

When Freigius worked out his text and this overview of liberal sciences in the 1570ies, psychology did not in fact come from nowhere. Hence, the quite recently discovered 1525 Castellani manuscript (Castellani 1525/2021), which is now translated into English (Janssen & Hubbard, 2021), is of extraordinary importance. The roots of this manuscript are most likely traceable back to 1512, but also to Gerhard Synellius. The text states, “the whole science about the soul [tota scientia de anima] […] is called Psychology [psychologia]” (Castellani 1525/2021, p.185). This statement of Castellani continues with saying, psychology “is said by the Greeks to be in the middle between Physics and Metaphysics”. This is an important statement for a couple of reasons. One is that the soul touches Metaphysics, but is not a part of it. The other is that the same is true for physics. This is partly in line with Aristotle, whose thesis *On the Soul* forms a background for his theory of knowledge, and consequently is a part of his philosophy (Klempe, 2020). Yet, the Castellani manuscript goes afar Aristotle by defining psychology explicitly, and as something that goes beyond both metaphysics and physics.

In this perspective, Castellani defines psychology as a science that combines those two types of knowledge. “Wherefore in this way the science about the soul [*scientia de anima*] is of a middle quality, just as mathematics and astronomy are of a middle quality” (Castellani 1525/2021, p.185).

To understand this comparison between mathematics and astronomy, it is necessary to bring in Hugh of St. Victor again. He already contrasted different qualities of mathematics by referring to per se (arithmetics), *al aliquid* (music), *immobilis* (geometry) and *mobilis* (astronomy). Most likely, however, the Platonic/Augustinian perspective of Hugh implied that the four where all regarded as pure mathematical sciences. Aristotle was not very much referred to when Hugh of St Victor lived. This was different when Castellani (and Synellius) were active. According to Aristotle, the soul acquires knowledge from both sensation and thinking. In this sense, the science of the soul is in the middle, as it is about the combination of physical sensation and pure thinking. When Castellani (and Synellis) use the term ‘quality’, it does not refer to the different types of mathematics, but instead to sense qualities. This is the radical change that appears in the sixteenth century. This is reflected in Freigius’ scheme, as he makes a fundamental distinction between the sciences of quantities and the sciences of qualities. Music and astronomy are not belonging to the mathematical sciences anymore, but to the sense-based experiential sciences. In this sense ‘physica’ in Freigius’ scheme is not referring to physics, but to what we would call physiology. And this is why psychology appears in the same category as music and astrology – they are all given through sensation as physically given experiences. Freigius’ scheme must be regarded as one of the earliest attempt to regard psychology as the science of sensation as such, as became the main content of *psychologia empirica*, which Christian Wolff included as a central part of the new and modern metaphysics he formed in the 1730ies.

## Conclusion

Although it is hard to define the subject of the history of psychology, there is no doubt that the term ‘psychology’ is a subject to pursue. When this is done, we see that the historiographical wisdom that should guide the research opens up for the multiplicity of the content of the term. Moreover, many aspects of this multiplicity are ignored and forgotten in the posterity. One of these is the aspect of music, which is highlighted in this paper, especially in connection with experimental psychology in the 19th Century. However, by pursuing this term, we have also achieved a more complete understanding of what changes the understanding of the soul went through during the sixteenth century. It was a turn from a mathematical to a sensational understanding of the soul. As the neologism ‘psychology’ appeared at the same time, this new term did not refer to the classic stability of mathematics, but to the dynamic aspects of sensation. This was a radical change, which opened up for the new worldview embedded in modernism in Europe.
